# Anti-IL17A Halts the Onset of Diabetic Retinopathy in Type I and II Diabetic Mice

**DOI:** 10.3390/ijms24021347

**Published:** 2023-01-10

**Authors:** Amy Y. Zhou, Brooklyn E. Taylor, Katherine G. Barber, Chieh A. Lee, Zakary R. R. Taylor, Scott J. Howell, Patricia R. Taylor

**Affiliations:** 1 Department of Ophthalmology and Visual Science, Case Western Reserve University School of Medicine, Cleveland, OH 44106, USA; 2 Louis Stokes Cleveland VA Medical Center, Cleveland, OH 44106, USA

**Keywords:** diabetes, diabetic retinopathy, IL-17A

## Abstract

There are ~463 million diabetics worldwide, and more than half have diabetic retinopathy. Yet, treatments are still lacking for non-proliferative diabetic retinopathy. We and others previously provided evidence that Interleukin-17A (IL-17A) plays a pivotal role in non-proliferative diabetic retinopathy. However, all murine studies used Type I diabetes models. Hence, it was the aim of this study to determine if IL-17A induces non-proliferative diabetic retinopathy in Type II diabetic mice, as identified for Type I diabetes. While examining the efficacy of anti-IL-17A as a potential therapeutic in a short-term Type I and a long-term Type II diabetes model; using different routes of administration of anti-IL-17A treatments. Retinal inflammation was significantly decreased (*p* < 0.05) after Type I-diabetic mice received 1 intravitreal injection, and Type II-diabetic mice received seven intraperitoneal injections of anti-IL-17A. Further, vascular tight junction protein Zonula Occludens-1 (ZO-1) was significantly decreased in both Type I and II diabetic mice, which was significantly increased when mice received anti-IL-17A injections (*p* < 0.05). Similarly, tight junction protein Occludin degradation was halted in Type II diabetic mice that received anti-IL-17A treatments. Finally, retinal capillary degeneration was halted 6 months after diabetes was confirmed in Type II-diabetic mice that received weekly intraperitoneal injections of anti-IL-17A. These findings provide evidence that IL-17A plays a pivotal role in non-proliferative diabetic retinopathy in Type II diabetic mice, and suggests that anti-IL-17A could be a good therapeutic candidate for non-proliferative diabetic retinopathy.

## 1. Introduction

Approximately 9.3% of the world population is diabetic [1]. Diabetes is typically categorized into two groups: Type I and Type II. Type I diabetes results from a chronic autoimmune disorder typically diagnosed in childhood or adolescence. Genetic predispositions or viral catalysts can trigger this autoimmune response against beta cells in the pancreas [2]. This causes immune cells and antibodies to destroy beta cells and inhibit insulin production [1]. Conversely, ~90% of all diabetics are Type II, which results from chronically elevated blood sugar levels [1]. Unlike Type I diabetes, the body is able to produce insulin. However, consistently high blood sugar levels lead to insulin resistance. Risk factors, such as obesity, can induce continuous increases in blood sugar that elicit insulin production. This can initiate beta cell malfunction and desensitization of the insulin receptors, which causes insulin resistance and hyperglycemia [1,3]. Although the activation mechanism of Type I and Type II diabetes is different, both lead to the same diabetic complications, such as diabetic retinopathy. Diabetic retinopathy is the most common microvascular diabetes complication, which causes blindness in ~10,000 diabetics each year [4]. Diabetes mediates microvascular lesions, hyperpermeability, and ischemia in the retina that can lead to vision impairment [5,6]. However, it is unclear if the same inflammatory pre-cursors that initiate diabetic retinopathy in Type I diabetics also induce retinopathy in Type II diabetics.

We and others have found that Interleukin-17A (IL-17A) is a prevalent inflammatory pre-cursor to diabetic retinopathy in Type I diabetic mice [7,8,9,10,11]. IL-17A is an inflammatory cytokine that mediates the severity of multiple autoimmune and inflammatory diseases, including Type I diabetes [12,13,14,15,16,17]. In our previous studies, we found that IL-17A played a pivotal role in the onset and progression of diabetic retinopathy in streptozotocin (STZ)-Type I-diabetic mice [7,8]. When IL-17A was systemically ablated in transgenic IL17A^−/−^ STZ-diabetic mice, the progression of retinal vascular impairment and the onset of diabetic retinopathy was halted [7,8]. In our mechanistic studies, we discovered that hyperglycemia activates transcription factor- RORγt (retinoic acid-related orphan receptor-γt) in circulating Th17 (Thelper-17) cells, which induces IL-17A production. Some of these circulating Th17 cells adhere to the retinal vasculature, break down the blood–retina barrier, and release IL-17A into the retina, which binds to the IL-17A receptor (IL-17R) on Muller glia and photoreceptors. After IL-17A binds to IL-17R, Act1-TRAF (TNF receptor associated factor) signaling cascades then activate NF-κB-dependent inflammation, FADD-dependent endothelial cell death, and ERK5-dependent oxidative stress in the diabetic retina. This leads to retinal vascular impairment and the onset of non-proliferative diabetic retinopathy [18,19,20,21].

Similarly, Qui et al. found that IL-17A played a pivotal role in retinal pathogenesis of STZ-induced and Akita Type I diabetic mice [9,10,11]. Unlike our findings, they concluded that retinal IL-17A was produced by Muller glia. Analogous to our findings, they determined that an IL-17R-Act1-TRAF6-NFκB signaling cascade in Muller glia played a pivotal role in retinal pathogenesis and the onset of non-proliferative diabetic retinopathy [9,10]. Notably, tight junction protein degradation and vascular leakage were halted 2 days after Type I diabetic mice received one intravitreal injection of a neutralizing anti-IL-17A or anti-IL-17RA antibody [10]. Considering this timeline, it is evident that these intravitreal injections were performed to determine the role of IL-17A in diabetic retinopathy. Yet, proof-of-principle that IL-17A could be a good therapeutic target for non-proliferative diabetic retinopathy was provided. This is very promising since there is an FDA approved, anti-IL-17A drug (secukinumab (i.e., Cosentyx)), which is currently used for treatment of psoriasis, psoriatic arthritis, and ankylosing spondylitis. Secukinumab is a human IgG1 monoclonal antibody that binds to IL-17A in vivo, and inhibits the binding of IL-17A to the IL-17A receptor. Hence, inhibiting IL-17A-dependent pathology in the same mechanistic manner as the intravitreal injections of anti-IL-17A or anti-IL-17RA [10]. Still, additional therapeutic studies are required to determine if anti-IL-17A could be a good therapeutic candidate for non-proliferative diabetic retinopathy.

Thus, the objective of our current study was to examine a mouse IgG1 monoclonal anti-IL-17A antibody (similar to Secukinumab) as a potential therapeutic in both Type I and II diabetic mice, while further examining multiple routes of administration, therapeutic concentrations, and elongated anti-IL-17A regimens. Notably, the findings define the role of IL-17A in Type II diabetes-induced non-proliferative diabetic retinopathy, while further defining the efficacy of anti-IL-17A as a potentially novel therapeutic for non-proliferative diabetic retinopathy.

## 2. Results

### 2.1. Clinical Data of STZ-Type 1 Diabetic Mice That Received an Intravitreal Injection of Anti-IL-17A

Per the American Diabetes Association, a hemoglobin A1C percentage (A1C) is a very reliable way to determine if an individual is diabetic, pre-diabetic, or non-diabetic [22]. An A1C level below 5.7% is considered non-diabetic, a level between 5.7 and 6.4% is considered pre-diabetic, and a level of 6.5% or higher is considered diabetic. These A1C levels are the same for diabetic mice. As shown in Table 1, all diabetic mice had an A1C above 6.5%, while the A1C for non-diabetic mice was below 5.7%. Further, A1C levels were significantly higher in both untreated and anti-IL17A-treated diabetic mice than their non-diabetic controls 6 weeks after diabetes was confirmed (and 1 week after the anti-IL-17A intravitreal injection). Additionally, there were no differences between anti-IL-17A-treated and -untreated diabetic mice. Body weight was also examined, whereas body weight of STZ-diabetic mice is normally lower than the non-diabetic controls [23]. As shown in Table 1, both the untreated and the anti-IL17A-treated diabetic mice displayed a significantly lower body weight than their non-diabetic controls, while there were no significant differences in the body weight of the anti-IL-17A-treated and -untreated diabetic mice.

### 2.2. Anti-IL-17A Intravitreal Injection Treatment in STZ-Type I Diabetic Mice

**Figure 1:**
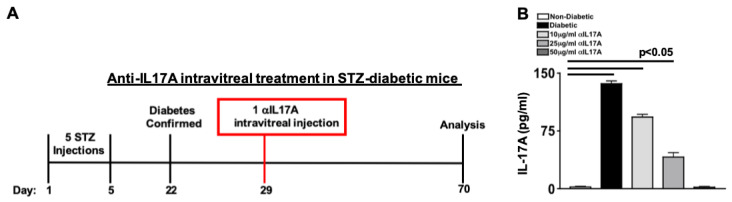
**Intravitreal Injection of anti-IL-17A in STZ-Type I Diabetic Mice.** (**A**) Schematic of STZ-Type I diabetic retinopathy murine model and anti-IL-17A intravitreal injection. (**B**) Quantification of IL-17A in retinas of non-diabetic, untreated diabetic, and 10 μg/mL, 25 μg/mL, and 50 μg/mL of anti-IL-17A treated diabetic mice; 1 week after one intravitreal injection. Error bars represent the SEM. The p-value was first equated by 2-way ANOVA analysis and then unpaired student’s *t*-test with Tukey’s post-hoc analysis.

To examine the efficacy of anti-IL17A treatment in retinal pathogenesis, a 1 μL intravitreal injection containing 10 μg/mL, 25 μg/mL, or 50 μg/mL of anti-IL17A monoclonal antibody was administered to STZ-diabetic mice, 1 week after diabetes was confirmed by a 6 h fasted blood glucose (FBG) greater than 275 mg/dL (Figure 1A). Three injected eyes of separate mice were pooled to make retinal protein lysates. Then, levels of IL-17A in three retinal protein samples per group was analyzed in triplicate wells by ELISA (1 week after αIL17A injection) to confirm IL-17A neutralization in the retina. As shown in Figure 1B, negligible levels of IL-17A was detected in the retina of non-diabetic mice, while ~140 pg/mL of IL-17A was detected in the retina of untreated diabetic mice. Approximately 85 pg/mL and ~35 pg/mL of IL-17A was detected in the retinas of mice that received 10 μg/mL and 25 μg/mL of anti-IL-17A, respectively. However, only negligible levels of IL-17A were detected in the diabetic mice that received one intravitreal injection of 50 μg/mL of anti-IL-17A. So, IL-17A is neutralized in the retina by one 50 μg/mL intravitreal injection of anti-IL-17A, and this concentration was used in all STZ-diabetic mice experiments.

### 2.3. Clinical Data of STZ-Diabetic Mice That Received a 50 μg/mL Intravitreal Injection of Anti-IL-17A

**Figure 2:**
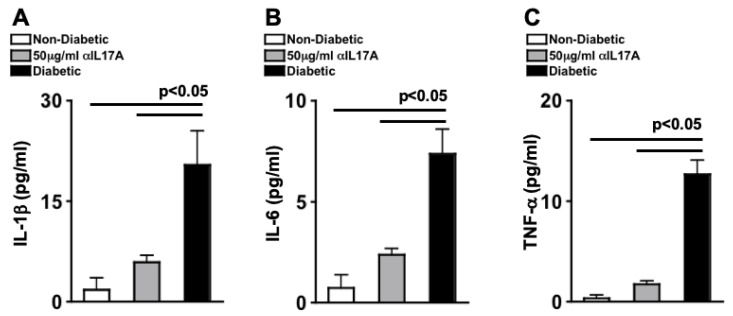
**Acute Phase Cytokines in the Retinas of STZ-Diabetic Mice After anti-IL-17A Injection.** Ella analysis of IL-1β (**A**), IL-6 (**B**), and TNF-α (**C**) in individual retinas (*n* = 9/group) of non-diabetic, anti-IL-17A treated, and untreated diabetic mice; 6 weeks after injection. Error bars represent the SEM. All p-values were first equated using 2-way ANOVA and then unpaired student’s *t*-test with Tukey’s post-hoc analysis. Data are representative of three separate experiments with similar results.

**Figure 3:**
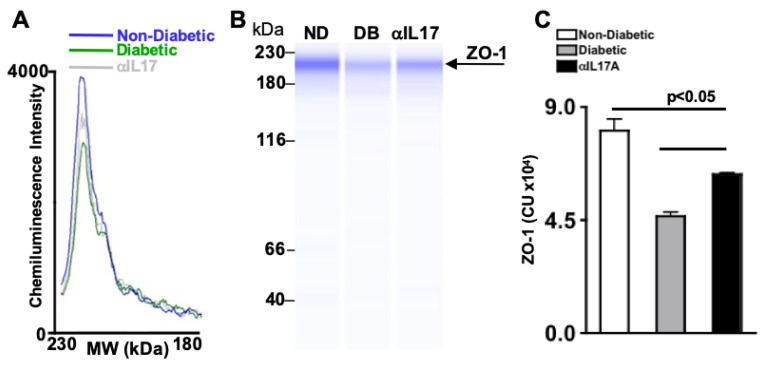
**ZO-1 Degradation in STZ-Diabetic Mice Receiving 1 anti-IL-17A Intravitreal Injection.** (**A**) Representative electropherogram of ZO-1 in retinas of non-diabetic, untreated STZ-diabetic, and IL-17A treated STZ-diabetic mice. (**B**) Representative Wes gel, and (**C**) quantification of ZO-1 in retinas of non-diabetic, anti-IL-17A treated diabetic, and untreated diabetic mice; 6 weeks after injection. Error bars represent the SEM. All p-values were first equated using 2-way ANOVA and then unpaired student’s *t*-test with Tukey’s post-hoc analysis. Data are representative of three separate Wes analyses from 3 experiments with similar results.

As shown in Table 2, hemoglobin A1C was examined in non-diabetic and STZ-diabetic C57BL/6 mice 6 weeks after intravitreal injection of 50 μg/mL of anti-IL-17A. Data are of two separate experiments, whereas 9 mice/group per experiment were examined. Non-diabetic mice were significantly lower than diabetic mice. Further, all non-diabetic mice had A1C scores below 5.7%, and all diabetic mice had A1C scores above 6.5%. This further confirms diabetic conditions. Both untreated and anti-IL-17A-treated STZ-diabetic mice had significantly lower body weight than the non-diabetic mice. Finally, there was no significant differences in A1C scores or body weight of the treated diabetic mice versus the untreated diabetic mice. Hence, diabetic conditions are confirmed for both treated and untreated diabetic mice examined in Figure 2 and Figure 3.

### 2.4. Anti-IL-17A Intravitreal Injection Decreases Retinal Inflammation in STZ-Diabetic Mice

The following experiment was triplicated in separate experiments. Individual retinas were collected to make retinal protein lysate samples. Three protein samples per group was collected ~6 weeks after anti-IL-17A intravitreal injection, for Ella (automated ELISA) quantification of IL-1β, IL-6, and TNF-α. Triplicate of each sample was analyzed, and the mean of each sample from all three experiments (*n* = 9/group) is displayed in Figure 2. There was an average of ~2 pg/mL of IL-1β (Figure 2A), ~1 pg/mL of IL-6 (Figure 2B), and ~1 pg/mL of TNF-α (Figure 2C) in the retinas of the non-diabetic mice, while ~25 pg/mL of IL-1β (Figure 2A), ~8 pg/mL of IL-6 (Figure 2B), and ~15 pg/mL of TNF-α (Figure 2C) were detected in the retinas of untreated STZ-diabetic mice, which was significantly decreased to ~5 pg/mL of IL-1β (Figure 2A), ~2 pg/mL of IL-6 (Figure 2B), and ~2 pg/mL of TNF-α (Figure 2C) in the retinas of diabetic mice that received an intravitreal injection of 50 μg/mL of anti-IL-17A. These data suggest that anti-IL-17A can decrease early stage retinal inflammation, which is a precursor to non-proliferative diabetic retinopathy. Further, the results suggest that 1 intravitreal injection of anti-IL-17A can provide protection against retinal inflammation 6 weeks post-injection.

### 2.5. Anti-IL17A Intravitreal Injection Halt ZO-1 Degradation in the Retinal Vasculature of STZ-Type I-Diabetic Mice

Diabetes can impair and degrade tight junction protein, zonula occludens-1 (ZO-1), in the retinal endothelium causing vascular permeability [24,25]. To determine if anti-IL-17A can inhibit ZO-1 degradation, three individual retinas were pooled in each retinal protein lysate. Protein of each sample was quantified by BCA (Bicinchoninic acid) analysis, and normalized so that equal amounts of samples were analyzed. Three samples of non-diabetic (blue), untreated diabetic (green), and anti-IL-17A-treated diabetic (grey) mice were analyzed by Wes (automated Western blot analysis).

Both chemiluminescence intensity (the peak height) and the amount of ZO-1 protein (the area under the curve) were examined by electropherogram (Figure 3A) and Wes capillary gels (Figure 3B). The amount of protein was then quantified in all samples and graphed in Figure 3C. As shown in Figure 3C, there is a significant decrease of ZO-1 in the retinas of untreated STZ-diabetic mice (grey) than non-diabetic mice (white). When STZ-diabetic mice received an intravitreal injection of anti-IL17A, a significant increase of ZO-1 was detected in the retina (black). Taken together, this suggests that anti-IL17A can halt ZO-1 degradation in the retina.

### 2.6. Clinical Data of Lepr^db^-Type II Diabetic Mice Receiving Weekly 10 μg/mL, 25 μg/mL, or 50 μg/mL of Anti-IL-17A Intraperitoneal Injections 2- and 6-Months Post-Diabetes

Hemoglobin A1C levels in all mice were examined 2- and 6-months after diabetes was confirmed. All diabetic mice (DB) had significantly higher A1C levels than non-diabetic mice (ND) (*n* = 9/group). Further, all non-diabetic mice had an A1C lower than 5.7%, which is in the non-diabetic range, and all diabetic mice had A1C scores higher than the 6.5% diabetic range. At 2 months, there were no differences between the anti-IL-17A-treated diabetic mice and untreated diabetic mice (Table 3). Yet, there was a slight (non-significant) decrease in hemoglobin A1C levels in all of the anti-IL-17A-treated versus the untreated diabetic mice. Further, all anti-IL-17A-treated and -untreated diabetic mice had a 6h fasted blood glucose concentration greater than 275 mg/dL; 2- and 6-months post-diabetes. Hence, all anti-IL-17A-treated and untreated diabetic mice were hyperglycemic at the times of analysis.

Lepr^db^ (db/db) mice spontaneously develop Type II diabetes associated with obesity and insulin resistance, so these diabetic mice are normally much larger than the non-diabetic controls. As shown in Table 3, all Lepr^db^ diabetic mice (DB) had a significantly higher body weight than the non-diabetic mice (ND). Further, there were no differences in body weight between any of the anti-IL-17A-treated and -untreated diabetic mice. Finally, to determine toxicity of anti-IL-17A injections, body weight measures, lethargy, mortality rate, respiratory stress, and autopsy organ appearance were examined in all treated non-diabetic mice. No toxicity was observed.

### 2.7. Anti-IL17A Treatment Regimen in Lepr^db^ Type II Diabetic Mice

**Figure 4:**
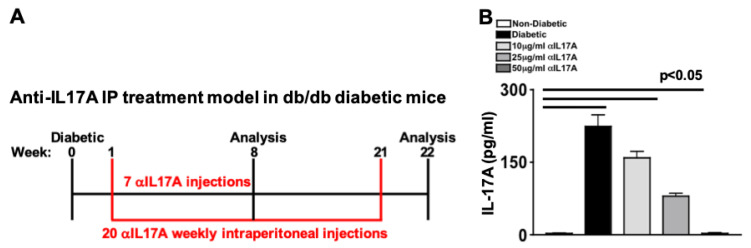
**Anti-IL-17A Treatment Regimen of db/db-Type II Diabetic Mice.** (**A**) Schematic of Lepr^db^-Type II-diabetic retinopathy murine model and anti-IL-17A intraperitoneal treatment regimen. (**B**) Quantification of IL-17A in retinas of non-diabetic, untreated diabetic, and treated diabetic mice with 10 μg/mL, 25 μg/mL, and 50 μg/mL of anti-IL-17A; 6 months post-diabetes. Error bars represent the SEM. The p-value was equated by 2-way ANOVA and then unpaired student’s *t*-test with Tukey’s post-hoc analysis.

To further examine the role of IL-17A in Type II diabetes-mediated diabetic retinopathy, and the efficacy of anti-IL-17A in Type II diabetic mice, an in vivo treatment regimen was designed in Lepr^db^ (db/db) diabetic mice. As shown in Figure 4A, weekly intraperitoneal injections of 100 μL of saline containing 10 μg/mL, 25 μg/mL, or 50 μg/mL of anti-IL17A was administered to non-diabetic (wild-type heterozygous Lepr^db^ mice) and Lepr^db^ Type II diabetic mice; 1 week after diabetes was confirmed with an FBG score greater than 275 mg/dL (Figure 4A). Levels of IL-17A in retinal protein lysates (*n* = 3 samples of 3 pooled retinas/group) were analyzed by ELISA 6 months post-diabetes (Figure 4B). Negligible levels of IL-17A was detected in the retina of non-diabetic mice (white), while ~240 pg/mL of IL-17A was detected in the retinas of untreated diabetic mice (black). Approximately 165 pg/mL and ~70 pg/mL of IL-17A was detected in the retinas of diabetic mice that received 10 μg/mL (light grey) and 25 μg/mL (dark grey) of anti-IL-17A, respectively. Similar to the non-diabetic mice, only negligible levels of IL-17A was detected in the retinas of diabetic mice that received 50 μg/mL of anti-IL-17A (slate grey). These data suggest that a weekly intraperitoneal injection of 50 μg/mL of anti-IL-17A was sufficient to neutralize IL-17A in the retina. Hence, 50 μg/mL of non-toxic, anti-IL-17A was administered weekly to mice that were hyperglycemic and later analyzed 2- and 6-months post-diabetes.

### 2.8. Clinical Data of db/db Mice Receiving Weekly 50 μg/mL of Anti-IL-17A Intraperitoneal Injections 2 Months Post-Diabetes

**Figure 5:**
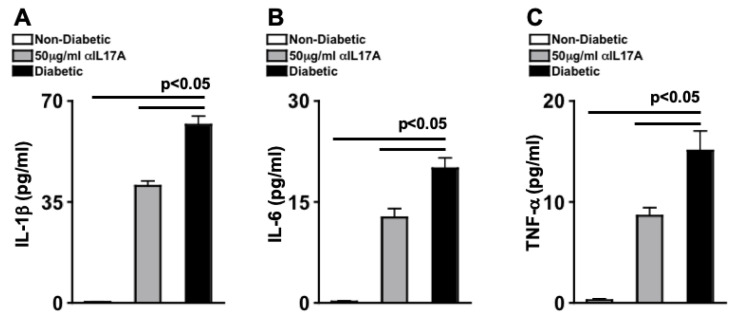
**Inflammatory Cytokines in the Retinas of db/db Mice Treated with anti-IL-17A.** Levels of IL-1β (**A**), IL-6 (**B**), and TNF-α (**C**) in protein lysates of individual retinas (*n* = 9/group) of non-diabetic, db/db diabetic treated with 50 μg/mL of anti-IL-17A, and untreated db/db diabetic mice; 2 months after diabetic conditions were confirmed. Error bars represent the SEM. All p-values were first equated using 2-way ANOVA and then unpaired student’s *t*-test with Tukey’s post-hoc analysis. Data are representative of three separate experiments.

**Figure 6:**
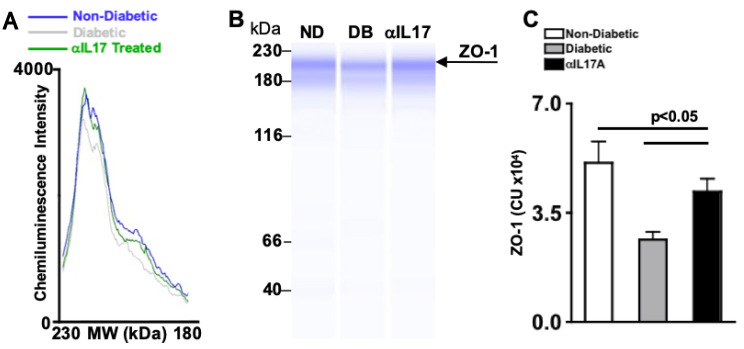
**ZO-1 Degradation in anti-IL-17A Treated db/db Mice 2 Months Post-Diabetes.** (**A**) Electropherogram of ZO-1 in retinal protein lysates of non-diabetic, untreated db/db diabetic, and anti-IL-17A treated db/db diabetic mice. (**B**) Representative Wes gel, and (**C**) protein quantification of ZO-1 in retinal protein lysates of non-diabetic (ND), untreated db/db diabetic (DB), and db/db diabetic mice treated with 50 μg/mL of anti-IL-17A; 2 months post-diabetes. Error bars represent the SEM. All p-values were equated using 2-way ANOVA and then unpaired student’s *t*-test with Tukey’s post-hoc analysis. Data are representative of three separate Wes analyses from with similar results.

As shown in Table 4, hemoglobin A1C was examined in non-diabetic Lepr^db^ heterozygous (HET) and Lepr^db^ (db/db) diabetic mice after receiving seven weekly intraperitoneal injections of 50 μg/mL of anti-IL-17A. Data are of two separate experiments, whereas nine mice/group per experiment were examined. A1C scores were significantly lower in non-diabetic mice than all diabetic mice. Further, all non-diabetic mice were in the A1C non-diabetic range, and all diabetic mice were in the A1C diabetic range, further confirming diabetic conditions. Additionally, all db/db diabetic mice had significantly higher body weight than the non-diabetic mice. Finally, there was no significant differences in A1C scores or body weight of the treated diabetic versus the untreated diabetic db/db mice. So, diabetes was confirmed for all mice examined in Figure 5 and Figure 6.

### 2.9. Anti-IL17A Treatments Significantly Decrease Inflammatory Cytokine Production in the Retina of Lepr^db^ Type II Diabetic Mice

Protein lysates of individual retinas (*n* = 9/group) were collected for automated Ella quantification of IL-1β, IL-6, and TNF-α; 2 months after diabetes was confirmed. As shown in Figure 5, only negligible levels of the pro-inflammatory cytokines were detected in the retinas of non-diabetic mice (white). In the retinas of untreated db/db diabetic mice (black), ~65 pg/mL (Figure 5A), ~20 pg/mL (Figure 5B), and ~18 pg/mL (Figure 5C) of IL-1β, IL-6, and TNF-α were detected, respectively. When the db/db diabetic mice received weekly intraperitoneal injections of anti-IL-17A treatments, IL-1β, IL-6, and TNF-α were significantly decreased.

### 2.10. Anti-IL17A Halts ZO-1 Degradation in db/db Diabetic Mice 2 Months Post-Diabetes

Wes analysis was run to detect and quantitate ZO-1. The representative electropherogram (Figure 6A) was plotted to display chemiluminescence versus molecular weight (MW), wherein the peak height corresponds to the intensity of a band shown in the gel (Figure 6B). The total amount of ZO-1 protein was calculated using the area under the curve of the electropherogram in all samples, and displayed in Figure 6C. As shown in Figure 6C, ZO-1 was significantly decreased in the retina of untreated diabetic mice (grey) than non-diabetic mice (white). Yet, there was a significant increase of ZO-1 in the retinas of anti-IL-17A-treated diabetic mice (black) when compared to untreated diabetic mice. These results provide evidence that anti-IL-17A halts the degradation of retinal ZO-1.

### 2.11. Clinical Data of Lepr^db^-Type II Diabetic Mice That Received Intraperitoneal Injections of 50 μg/mL of Anti-IL-17A 6 Months after Diabetic Conditions Were Confirmed

**Figure 7:**
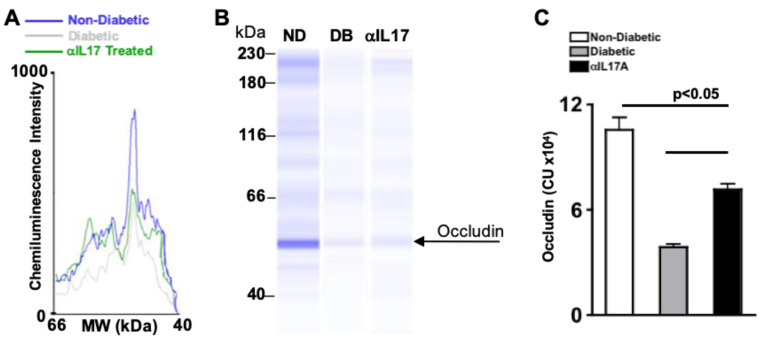
**Occludin Degradation in anti-IL-17A treated db/db Diabetic Mice 6 Months Post-Diabetes.** (**A**) Representative electropherogram of Occludin in retinal protein lysates of non-diabetic (blue), untreated db/db diabetic (grey), and anti-IL-17A treated db/db diabetic (green). (**B**) Wes gel of Occludin in retinal protein lysates of non-diabetic (ND), untreated db/db diabetic (DB), and db/db diabetic mice treated with 50 μg/mL of anti-IL-17A (αIL17); 6 months post-diabetes. (**C**) Occludin protein quantification in retinas of non-diabetic (white), untreated db/db diabetic (grey), and db/db diabetic mice treated with intraperitoneal injections of anti-IL-17A (black). Error bars represent the SEM. All p-values were first equated using 2-way ANOVA and then unpaired student’s *t*-test with Tukey’s post-hoc analysis. Data are represenetative of 3 separate Wes analyses.

**Figure 8:**
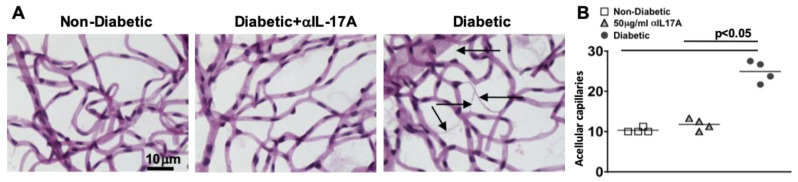
**Retinal Capillary Degeneration in anti-IL-17A Treated db/db Mice.** (**A**) Representative images of acellular capillaries (highlighted by black arrows) in retinal capillary beds of non-diabetic, anti-IL-17A treated diabetic, and untreated db/db diabetic mice (scale bars of all images = 10 μm. (**B**) Quantification of acellular capillaries in each retina of non-diabetic, anti-IL-17A treated diabetic, and untreated diabetic mice; 6 months post-diabetes. Each data point represents an individual retina. Error bars represent the SEM. All *p*-values were first equated using 2-way ANOVA and then unpaired student’s *t*-test with Tukey’s post-hoc analysis.

Hemoglobin A1C was measured in non-diabetic Lepr^db^ heterozygous (HET) and Lepr^db^ (db/db) diabetic mice after receiving 20 weekly intraperitoneal injections of 50 μg/mL of anti-IL-17A (Table 5). Data are of two separate experiments, whereas a total of 14 mice/group were examined. A1C scores were significantly lower in non-diabetic heterozygous controls than all diabetic mice. All non-diabetic mice had an A1C score below 5.7% and all diabetic mice had an A1C score above 6.5%. Further, all db/db diabetic mice had significantly higher body weight than the non-diabetic heterozygous controls. Yet, there was no significant differences in A1C scores or body weight between the anti-IL-17A-treated diabetic and the untreated diabetic mice. These mice were further examined in experiments displayed in Figure 7 and Figure 8.

### 2.12. Anti-IL17A Treatments Halt Occludin Degradation in db/db Type II Diabetic Mice; 6 Months Post-Diabetes

Wes analysis quantitated tight junction protein Occludin in normalized protein lysates of three pooled retinas from non-diabetic heterozygous controls, untreated and anti-IL-17A-treated db/db diabetic mice. Chemiluminescence intensity of the Occludin between 66 and 40 kDa in retinal protein lysates is displayed in the electropherogram (Figure 7A). The peak height correlates to the Wes band intensity of Occludin shown in the gel (Figure 7B). The total amount of Occludin protein detected in three different samples was calculated using the area under the curve of the electropherogram, and graphed in Figure 7C. As shown in Figure 7C, Occludin was significantly decreased in the retina of untreated db/db diabetic mice (grey) when compared to non-diabetic heterozygous controls (white). However, Occludin was significantly increased in the retinas of db/db diabetic mice that received weekly intraperitoneal injections of anti-IL-17A (black). These results provide evidence that intraperitoneal injections of anti-IL-17A halts the degradation of Occludin in the retina of Type II db/db mice; 6 months after diabetes was confirmed.

### 2.13. Anti-IL17A Treatments Halts Capillary Degeneration and the Early Onset of Diabetic Retinopathy in Lepr^db^ Type II Diabetic Mice 6 Months Post-Diabetes

Lepr^db^ mice develop capillary degeneration and non-perfusion, which is a clinical hallmark of early stage non-proliferative diabetic retinopathy [26]. To ascertain if the weekly anti-IL-17A treatments can halt non-proliferative diabetic retinopathy, retinal capillary degeneration was examined 6 months after diabetes was confirmed. Acellular capillaries in the retinas of non-diabetic, anti-IL-17A-treated and untreated diabetic mice (*n* = 5/group) were quantified (representative examples are highlighted by black arrows in Figure 8A). The number of acellular capillaries in the retinas of diabetic mice was significantly higher than in non-diabetic mice (Figure 8B). However, when the diabetic mice were treated with anti-IL-17A, the number of acellular capillaries was significantly decreased to similar numbers of acellular capillaries in non-diabetic mice (Figure 8B). Hence, weekly 50 μg/mL anti-IL-17A treatments can halt the retinal pathogenesis and vascular impairment that is observed in early stage non-proliferative diabetic retinopathy.

## 3. Discussion

Overall, this study provides evidence that one 50 μg/mL intravitreal injection of anti-IL-17A significantly decreased (*p* < 0.05) inflammatory cytokines: IL-1β, IL-6, and TNF-α in the retina of STZ-Type I- diabetic mice. Additionally, an anti-IL-17A intravitreal injection was capable of halting diabetes-mediated ZO-1 degradation 6 weeks after injection. Similarly, weekly intraperitoneal injections of 50 μg/mL of anti-IL-17A significantly decreased (*p* < 0.05) diabetes-mediated IL-1β, IL-6, TNF-α, and ZO-1 degradation in the retinas of db/db-Type II diabetic mice 2 months post-diabetes. Finally, weekly intraperitoneal injections of 50 μg/mL of anti-IL-17A halted Occludin degradation and capillary degeneration of the retina in db/db-Type II-diabetic mice; 6 months after diabetic conditions was confirmed. Since this vascular impairment is a hallmark of non-proliferative diabetic retinopathy, these results suggest that anti-IL-17A could be a potentially novel therapeutic for early stage diabetic retinopathy in both Type I and II diabetics.

In both Type I and II diabetes, inflammation plays a pivotal role in the onset and progression of diabetic retinopathy. In Type I diabetes, neutrophils, autoimmune-driven macrophages, T cells, and natural killer cells continuously produce low levels of inflammatory cytokines, while in Type II diabetes, obesity triggers adipose cell leakage that induces chronic low-grade inflammation. Over time, chronic low-grade inflammation can breakdown the blood–retina barrier, immune cells and cytokines can enter the retina, which can induce retinal inflammation. This can then lead to vascular impairment and the onset of diabetic retinopathy [27]. We and others have found that IL-17A is one of the pivotal inflammatory components that induces retinal pathogenesis, vascular impairment, and the onset of diabetic retinopathy [7,8,28,29,30].

Byrne et al. determined that tight junction protein-ZO-1 was degraded when human retinal pigment endothelial cells were incubated with recombinant IL-17A [28]. Similarly, in our current study we found a significant decrease in retinal ZO-1 in both Type I and II diabetic mice, which was significantly increased when diabetic mice were treated with anti-IL-17A. ZO-1 is a tight junction protein that helps form the barriers of epithelial cells and increased trans epithelial electrical resistance, which controls vascular permeability in the retina [29]. Similarly, Occludin is another tight junction protein that can control vascular permeability, and is degraded in the diabetic retina [30]. Both Occludin and ZO-1 were degraded in Type I and II diabetic mice. Yet, anti-IL-17A treatments significantly decreased the degradation of these vascular tight junction proteins, which are precursors to vascular impairment in the retina. The efficacy of anti-IL-17A in delaying vascular impairment was further solidified, when capillary degeneration was halted in the db/db Type II diabetic mice that received weekly intraperitoneal injections of anti-IL-17A. Collectively, this provides strong evidence that IL-17A plays a pivotal role in Type II diabetes induced diabetic retinopathy. Further suggesting that anti-IL-17A could be a potentially novel therapeutic for non-proliferative diabetic retinopathy.

Previously, we and others have found that IL-17A production is induced in multiple Type I diabetes murine models. Further, significant levels of IL-17A were detected in the retina [7,8,9,10,11]. Additionally, when IL-17A was systemically ablated in STZ-induced diabetic IL17A^−/−^ mice, acute phase cytokine production, vascular leakage, and capillary degeneration in the retina was ameliorated [7,8]. Our previous studies provided evidence that diabetes induced immune cells to produce IL-17A that migrated through the retinal vasculature, induced retinal inflammation, and vasculature impairment. Yet, Qui et al. determined that hyperglycemia induced Muller glia to produce IL-17A. Hence, it was unclear if a monoclonal antibody that is too large to cross the blood–retina barrier (like anti-IL-17A) would have to be administered via an intravitreal injection, like the current anti-VEGF treatments. Conversely, if systemically migrating immune cells were the source of IL-17A, anti-IL-17A could be administered via a less intrusive route, such as the intraperitoneal injection regimen that we performed. The results of this current study provide evidence that anti-IL-17A could be administered via a systemic route and still halt the onset and progression of diabetic retinopathy. This is therapeutically advantageous, since IL-17A plays a pathologic role in other diabetic complications, such as nephropathy [31]. Hence, it is possible that anti-IL-17A could be used to halt the progression of all diabetic complications if administered systemically as we did in the treatment regimen in the Lepr^db^ diabetic mice.

One of the most prevalent treatments for late-stage diabetic retinopathy is intravitreal injections of anti-VEGF [6]. VEGF is primarily responsible for the growth of new blood vessels throughout the body. Due to the systemic prevalence of VEGF, anti-VEGF drugs are currently used to treat different types of cancers (especially renal cancer), wet age-related macular degeneration, and macular edema, as well as diabetic retinopathy [32,33,34,35,36]. Anti-VEGF treatments of diabetic macular edema normally halt the progression of neovascularization and diabetic retinopathy. However, anti-VEGF treatments are not effective in early stage mild to moderate non-proliferative diabetic retinopathy. Additionally, ~40% of patients with proliferative diabetic retinopathy or diabetic macular edema do not respond to anti-VEGF treatments [6,37,38]. This same type of phenomenon has been observed in cancer, wherein cancer patients do not respond to anti-VEGF treatments [39]. Previous cancer studies provide evidence that anti-VEGF resistance is driven by tumor-secreted IL-17A [39,40,41]. When anti-IL-17A was administered prior to anti-VEGF treatment, antitumor activity and the efficacy of anti-VEGF treatments was restored [40]. Our future studies will focus on a combined treatment of anti-IL-17A and anti-VEGF in late-stage diabetic retinopathy and neovascularization. This will help us further determine if anti-IL-17A could be a potentially novel therapeutic for both early and late stage diabetic retinopathy.

There were a few limitations to this anti-IL-17A therapeutic study. First, we only performed one intravitreal injection in the STZ-diabetic mice. Further long-term studies with multiple intraperitoneal injections need to be performed to examine the clinical relevance of anti-IL-17A as a therapeutic. However, this might have to be performed in a larger animal than mice because it is unclear if multiple intravitreal injections would be safe for mice. Additionally, more elaborate pre-clinical experiments are needed to further clarify the validity of anti-IL-17A as a therapeutic for diabetic retinopathy, which goes beyond the scope of this paper but will be the focus of our future studies.

In conclusion, several notable discoveries emerged in this study. First, we provide evidence that IL-17A plays a similar pathologic role in Type II diabetes-induced diabetic retinopathy as previously identified in Type I diabetes. Further, anti-IL-17A administered systemically and via intravitreal injection was sufficient to halt retinal inflammation and tight junction protein degradation. Finally, we designed an anti-IL-17A treatment regimen in a murine model of Type II diabetes that halted diabetes-mediated retinal inflammation, vascular impairment, and the onset of diabetic retinopathy. Taken together, we further validate that anti-IL-17A could be a potentially novel therapeutic for non-proliferative diabetic retinopathy in both Type I and Type II diabetics.

## 4. Materials and Methods

### 4.1. C57BL/6 Streptozotocin (STZ)-Induced Diabetic Mice

Mice (6–8 weeks of age) were injected with 60 mg/Kg BW streptozotocin (STZ) (MP Biomedicals, Solon, OH, USA) in 0.1M citrate buffer (pH 4.5) IP for five consecutive days, each after 6h fast. Food was returned to the mice immediately after each day’s injection. Other than 6h fastings, water and food was provided ad lib throughout the length of each study. Diabetic status was defined by 6h fasted blood glucose (FBG) concentrations greater than 275 mg/dL verified on three instances between days 14 to 21 post-last-STZ injection, using conventional consumer glucose testing meters/strips (non-diabetic mice typically have FBG values of 150 ± 40 mg/dL). Hyperglycemia severity and diabetic conditions were quantified using hemoglobin A1C via Crystal Chem Mouse A1c kit and Controls (Elk Grove Village, IL, USA). A1C scores below 5.7% is considered non-diabetic, and A1C scores above 6.5% is considered diabetic [22]. Mice are weighed weekly and 0 to 0.2 Units of insulin (Humulin N, NPH, Eli Lilly, Indianapolis, IN, USA) therapy was administered as-needed to maintain proper body weight.

### 4.2. Anti-IL17A Intravitreal Injection

Mice were anesthetized using IP injection of Ketamine: Xylazine cocktail, after which proparacaine was used to further anesthetize the eye. Tropicamide (1% sterile ophthalmic solution, USP) was then applied to the procedure eye to dilate the pupil. A beveled 34-gauge needle (NanoFil, World Precision Instruments, Sarasota, FL, USA) was inserted perpendicular to the surface of the globe at ~2 mm posterior to the limbus to directly reach the vitreous cavity while minimizing possible damage to the physical structures of the eye including the lens, central retina, and optic nerve. The sharp needle was then slowly withdrawn from the eye and immediately replaced with a blunt-tip 34-gauge needle, which was inserted into the eye via the hole. A 1 μL volume of 10 μg/mL, 25 μg/mL, or 50 μg/mL anti-mouse IL-17A (a neutralizing, monoclonal IgG1 antibody that binds to IL-17A, and inhibits IL-17A activity) antibody (BioXCell, Lebanon, NH, USA) was delivered intravitreal via micro syringe (Sub-Microliter Injection System, World Precision Instruments, Sarasota, FL). The needle was held in place for 10s after injection and then slowly removed in order to insure no fluid leaks from the globe. Both eyes were then covered with GenTeal 0.3% Hypromellose gel (Alcon, Fort Worth, TX, USA) to protect the corneas from drying and animals recovered in a warmed post-surgical recover chamber. Ophthalmic bacitracin-neomycin-polymyxin triple antibiotic ointment was applied to the procedure eye once daily for 3 days to prevent infection.

### 4.3. IL-17A ELISA Analysis

Retinal protein lysates were collected from db/db or C57BL/6 mice, and analyzed for IL-17A protein using an ELISA according to the manufacturer’s directions (R&D Biosciences, Minneapolis, MN, USA). These analyses were used to confirm IL-17A neutralization. When the proper concentration of anti-IL-17A is administered, it will bind to all IL-17A. When IL-17A is bound to anti-IL-17A, it is inhibited from binding to the capture antibody of the ELISA. Hence, IL-17A will not be detected by ELISA when sufficiently neutralized by anti-IL-17A.

### 4.4. Ella Automated Immunoassay Analysis

Samples were solubilized in 2x lysis buffer (RayBiotech, Peach Tree Corners, GA, USA). Following BCA protein quantification (Pierce, Waltham, MA, USA), samples were normalized and loaded onto Ella (automated ELISA) plates coated with anti-mouse IL-1β, IL-6, and TNF-α antibody (Protein Simple, Biotechne, Minneapolis, MN, USA). Manufacturer’s instructions were followed and results are displayed in Figure 2 and Figure 5.

### 4.5. Wes Automated Western Blot Analysis

Retinal tissue samples were solubilized in RIPA buffer (Pierce, Waltham, MA, USA) containing proteolytic inhibitor cocktail (Thermo Scientific, Waltham, MA, USA). Samples were quantified using a BCA assay (Pierce, Waltham, MA, USA). Protein samples were normalized, whereas equal amounts of protein in each sample was loaded onto a WES (automated Western) cartridge (Protein Simple, Biotechne, Minneapolis, MN, USA). Samples were run according to manufacturer’s instructions. Anti-ZO-1 and anti-Occludin (DSHB, Iowa City, IA, USA) antibodies were used for detection. Results are displayed in Figure 3, Figure 6 and Figure 7.

### 4.6. Lepr^db^ (db/db) Diabetic Mice

Mice homozygous for the *Lepr^db^* spontaneous diabetes mutation (Jackson Laboratory, Bar Harbor, ME, USA) were used to model Type II diabetes. Animal weights were monitored weekly. Diabetic status was determined from repeated weekly 6h fasted blood glucoses upon arrival of mice from vendor and acclimation to our facility. Anti-mouse IL-17A neutralizing monoclonal antibody (BioXCell, Lebanon, NH, USA) was injected once per week IP, starting one week following confirmation of diabetic status, for a total of 7 injections for short-term experiments (mice analyzed 2 months post-diabetes), and 20 injections for long-term experiments (mice analyzed 6 months post-diabetes). Heterozygous mice were used as non-diabetic controls.

### 4.7. Anti-IL17A Intraperitoneal Treatment Regimen

Mice were manually restrained and given intraperitoneal injections of 10, 25 or 50 μg/mL of anti-mouse IL-17A neutralizing antibody (BioXCell, Lebanon, NH, USA), weekly, using 25-gauge needle, alternating injection site every week to contralateral from previous week to prevent injection site reactions.

### 4.8. Retinal Capillary Degeneration

Acellular capillaries were quantified in the retinal vasculature as previously described [18,19,20,21]. Eyes were fixed with 10% formalin. Retinas were incubated in elastase for 2h followed by acidic buffer overnight. Retinal vasculature was stained with hematoxylin and periodic acid-Schiff. Acellular capillaries were quantified in 7 field areas between the optic nerve and the periphery (200× magnification).

### 4.9. Statistical Analysis

Statistical analysis was performed using a two-way ANOVA analysis and an unpaired *t*-test with Tukey’s post hoc analysis (Prism version 9, GraphPad Software, San Diego, CA, USA). A *p*-value < 0.05 was considered significant.

## Figures and Tables

**Table 1:** Clinical data of STZ-diabetic mice receiving intravitreal injection of anti-IL-17A.

Group	Number of Mice	HbA_1C_ (%)	Body Weight (g)
C57BL/6 ND-untreated	*n* = 9	4.5 ± 0.3	36.8 ± 0.8
C57BL/6 STZ-DB-untreated	*n* = 9	12.1 ± 1.3 *	26.1 ± 2.1 *
αIL17A (10 μg/mL) C57BL/6 ND	*n* = 9	5.1 ± 0.4	31.7 ± 0.6
αIL17A (10 μg/mL) C57BL/6 STZ-DB	*n* = 9	11.8 ± 1.4 *	24.1 ± 1.1 *
αIL17A (25 μg/mL) C57BL/6 ND	*n* = 9	4.8 ± 0.5	33.1 ± 0.6
αIL17A (25 μg/mL) C57BL/6 STZ-DB	*n* = 9	11.1 ± 1.8 *	22.0 ± 1.5 *
αIL17A (50 μg/mL) C57BL/6 ND	*n* = 9	4.7 ± 0.3	35.2 ± 0.9
αIL17A (50 μg/mL) C57BL/6 STZ-DB	*n* = 9	12.2 ± 1.1 *	23.3 ± 0.5 *

Data are mean ± SD. * = *p* < 0.01 diabetic (DB) compared to non-diabetic (ND) per group.

**Table 2:** Clinical data of STZ-diabetic mice receiving intravitreal injection of anti-IL-17A.

Group	Number of Mice	HbA_1C_ (%)	Body Weight (g)
C57BL/6 ND-untreated	*n* = 18	4.7 ± 0.2	33.2 ± 0.3
C57BL/6 STZ-DB-untreated	*n* = 18	11.7 ± 0.6 *	24.3 ± 1.1 *
αIL17A (50 μg/mL) C57BL/6 STZ-DB	*n* = 18	11.2 ± 1.0 *	23.8 ± 0.9 *

Data are mean ± SD of 3 separate experiments. * = *p* < 0.01 diabetic (DB) compared to non-diabetic (ND) per group.

**Table 3:** Clinical data of db/db diabetic mice receiving intraperitoneal injections of anti-IL-17A.

Group	HbA_1C_ (%) Week 6	Weight (g) Week 6	HbA_1C_ (%) Week 22	Weight (g) Week 22
Lepr^db^ Het ND-untreated	4.4 ± 0.3	32.7 ± 6.4	4.2 ± 0.1	39.3 ± 1.5
Lepr^db^ DB-untreated	11.2 ± 0.7 *	66.3 ± 3.1 *	12.7 ± 1.4 *	73.0 ± 6.3 *
αIL17A (10 μg/mL) Lepr^db^ Het ND	4.8 ± 0.4	27.7 ± 0.6	4.2 ± 0.8	28.7 ± 1.2
αIL17A (10 μg/mL) Lepr^db^ DB	10.7 ± 0.8 *	66.0 ± 5.3 *	11.9 ± 1.8 *	71.7 ± 2.3 *
αIL17A (25 μg/mL) Lepr^db^ Het ND	4.4 ± 0.4	32.0 ± 2.3	4.3 ± 0.6	29.3 ± 3.2
αIL17A (25 μg/mL) Lepr^db^ DB	11.4 ± 0.7 *	65.0 ± 1.6 *	10.7 ± 0.5 *	70.3 ± 6.7 *
αIL17A (50 μg/mL) Lepr^db^ Het ND	4.4 ± 0.1	39.0 ± 4.0	4.2 ± 0.3	29.3 ± 2.1
αIL17A (50 μg/mL) Lepr^db^ DB.	11.5 ± 0.5 *	64.0 ± 5.4 *	11.9 ± 0.3 *	67.7 ± 6.8 *

Data are mean ± SD. * = *p* < 0.01 diabetic (DB) compared to non-diabetic (ND) per group, whereas *n* = 9 mice/group.

**Table 4:** Clinical data of db/db mice receiving weekly intraperitoneal injection of 50 μg/mL of anti-IL-17A 2 months post-diabetes.

Group	Number of Mice	HbA_1C_ (%)	Body Weight (g)
Lepr^db^ Het ND-untreated	*n* = 18	4.3 ± 0.4	38.9 ± 2.3
Lepr^db^ (db/db) DB-untreated	*n* = 18	12.2 ± 0.9 *	64.1 ± 4.1 *
αIL17A (50 μg/mL) Lepr^db^ (db/db) DB	*n* = 18	11.5 ± 0.5 *	65.8 ± 0.9 *

Data are mean ± SD of 3 separate experiments. * = *p* < 0.01 diabetic (DB) compared to non-diabetic (ND) per group.

**Table 5:** Clinical data of db/db mice receiving weekly intraperitoneal injection of 50 μg/mL of anti-IL-17Al 6 months post-diabetes.

Group	Number of Mice	HbA_1C_ (%)	Body Weight (g)
Lepr^db^ Het ND-untreated	*n* = 14	4.3 ± 0.7	38.9 ± 2.3
Lepr^db^ DB-untreated (db/db)	*n* = 14	12.8 ± 1.3 *	64.1 ± 4.1 *
αIL17A (50 μg/mL) Lepr^db^ DB (db/db)	*n* = 14	11.9 ± 1.1 *	65.8 ± 0.9 *

Data are mean ± SD of 2 separate experiments. * = *p* < 0.01 diabetic (DB) compared to non-diabetic (ND) per group.

## Data Availability

The dataset generated during and/or analyzed during the current study are available from the corresponding author on reasonable request.

## Abbreviations

αIL-17A	anti-Interleukin-17A antibody
A1C	Hemoglobin glycated average blood sugar
ANOVA	Analysis of variance
Act1	adaptor molecule 1
BCA	Bicinchoninic acid
BW	Body weight
CU	Chemiluminescent unit
DB	Diabetic
db/db	Lepr^db^ mice
ELISA	Enzyme-linked immunosorbent assay
ERK5	Extracellular signal receptor kinase 5
FADD	Fas-associated death domain
FBG	Fasted blood glucose
FDA	Food and drug administration
Het	Heterozygous
IgG1	Immunoglobulin G1
IL-1β	Interleukin-1 beta
IL-6	Interleukin-6
IL-17A	Interleukin-17A
IL17R	Interleukin-17A receptor
IL-17RA	Interleukin-17A receptor A
IP	Intraperitoneal
kDa	Kilo-Dalton
MW	Molecular weight
ND	Non-diabetic
NFκB	Nuclear factor kappa B
RORγt	Retinoic acid-related orphan receptor-gamma t
SD	Standard deviation
SEM	Standard error of the mean
STZ	Streptozotocin
Th17	T helper 17
TNF-α	Tumor necrosis factor alpha
TRAF	TNF receptor associated factor
TRAF6	TNF receptor associated factor 6
VEGF	Vascular endothelial growth factor
ZO-1	Zonula occludens-1
